# Morphine drives internal ribosome entry site-mediated hnRNP K translation in neurons through opioid receptor-dependent signaling

**DOI:** 10.1093/nar/gku1016

**Published:** 2014-10-31

**Authors:** Pin-Tse Lee, Po-Kuan Chao, Li-Chin Ou, Jian-Ying Chuang, Yen-Chang Lin, Shu-Chun Chen, Hsiao-Fu Chang, Ping-Yee Law, Horace H. Loh, Yu-Sheng Chao, Tsung-Ping Su, Shiu-Hwa Yeh

**Affiliations:** 1 Institute of Biotechnology and Pharmaceutical Research, National Health Research Institutes, Miaoli County 35053, Taiwan, ROC; 2 The PhD Program for Neural Regenerative Medicine, Taipei Medical University, Taipei 110, Taiwan, ROC; 3 Graduate Institute of Biotechnology, Chinese Culture University, Taipei 11114, Taiwan, ROC; 4 Department of Pharmacology, University of Minnesota, Medical School, Minneapolis, MN 55455, USA; 5 Cellular Pathobiology Section, Intramural Research Program, National Institute on Drug Abuse, Baltimore, MD 21224, USA

## Abstract

Heterogeneous nuclear ribonucleoprotein K (hnRNP K) binds to the promoter region of mu-opioid receptor (MOR) to regulate its transcriptional activity. How hnRNP K contributes to the analgesic effects of morphine, however, is largely unknown. We provide evidence that morphine increases hnRNP K protein expression via MOR activation in rat primary cortical neurons and HEK-293 cells expressing MORs, without increasing mRNA levels. Using the bicistronic reporter assay, we examined whether morphine-mediated accumulation of hnRNP K resulted from translational control. We identified potential internal ribosome entry site elements located in the 5′ untranslated regions of hnRNP K transcripts that were regulated by morphine. This finding suggests that internal translation contributes to the morphine-induced accumulation of hnRNP K protein in regions of the central nervous system correlated with nociceptive and antinociceptive modulatory systems in mice. Finally, we found that down-regulation of hnRNP K mediated by siRNA attenuated morphine-induced hyperpolarization of membrane potential in AtT20 cells. Silencing hnRNP K expression in the spinal cord increased nociceptive sensitivity in wild-type mice, but not in MOR-knockout mice. Thus, our findings identify the role of translational control of hnRNP K in morphine-induced analgesia through activation of MOR.

## INTRODUCTION

Opioids are the most effective analgesics used clinically to alleviate moderate to severe pain (1). Among the opioids, morphine is a mainstay of modern pain management (2). Opioid receptors belong to the G protein-coupled receptor (GPCR) family, have site-specific effects and are expressed mainly in the central nervous system (CNS) (3). Three subtypes of opioid receptors exist in the mammalian brain: the mu, kappa and delta receptors. These receptors mediate the analgesic effects of opioids via several receptor–effector mechanisms (4). In the nervous system, opioids and their analogs bind with opioid receptors, which subsequently activate potassium channels and inhibit cyclic adenosine 3′,5′-phosphate (cAMP) production (5). The mu-opioid receptor (MOR) is crucial to the analgesic and reward-related effects *in vivo* because the antinociceptive effect of morphine is totally abolished in MOR knockout (KO) mice (6). Although morphine is a potent drug widely used for clinical treatments, the regulation mechanisms and signaling pathways that mediate morphine-induced analgesia are not well concluded.

Heterogeneous nuclear ribonucleoprotein K (hnRNP K) belongs to the poly(C) binding protein family, contains three KH (hnRNP K homology) domains, which plays a critical role in its binding affinity. hnRNP K comprises an hnRNP K-specific nuclear shuttling domain and nuclear localization signal sequence that regulates protein transport from the cytoplasm to the nucleus (7). hnRNP K is a ubiquitous, multi-functional RNA binding protein (RBP) that is involved in RNA processing, chromatin remodeling, transcription, translation and mRNA turnover (8). As a transcription factor, hnRNP K modulates tumor growth by regulating tumor-related proteins, such as the oncogenes c-myc, c-src and BRCA1 (9–11). Nevertheless, how hnRNP K functions in the nervous system is still poorly understood.

Translation initiation plays a major role in the regulation of protein synthesis. Translational control includes two regulation mechanisms: a cap-dependent process and a cap-independent mechanism involving ribosome binding to an internal ribosome entry site (IRES) (12). A previous report demonstrated that hnRNP K can bind to the promoter region of mouse MOR (mMOR) and act as an activator to modulate transcription in neuronal cells (13). Thus, hnRNP K may play a role of MOR-mediated functions, such as analgesic effects induced by morphine. In the present study, we examined the functions of hnRNP K by determining its protein expression levels in the mouse brain and spinal cord. We used the human embryonic kidney 293 (HEK-293) cells constitutively expressing MOR (HEK-MOR) and the tail-flick test of mice to evaluate the regulatory mechanisms of hnRNP K that modulate morphine-mediated analgesia.

## MATERIALS AND METHODS

### Cell culture

HEK-MOR cells (kindly provided by Dr. Ping-Yee Law, University of Minnesota, USA) were cultured in high-glucose Dulbecco's modiﬁed Eagle's medium (DMEM, GIBCO) supplemented with 400 μg/ml G418 (Sigma), 2mM L-glutamine and P/S/F (100 units/ml penicillin, 100 μg/ml streptomycin, 10% fetal bovine serum). Mouse pituitary AtT-20 cells were cultured in DMEM containing P/S/F. Mouse neuroblastoma Neuro-2a cells were cultured in minimum essential media (GIBCO) containing P/S/F. Rat primary cortical neurons were cultured in Neurobasal-A medium (GIBCO) supplemented with 2 mM l-glutamine and P/S/F. The cultures were incubated at 37°C in a humidified 5% CO_2_ incubator.

### Plasmid constructions, short interfering RNA (siRNA) and reporter assays

The primers used for polymerase chain reaction (PCR) amplification and plasmid constructions were listed in Supplementary Table S1. The bicistronic reporter constructs containing expression of *Renilla* luciferase and *Firefly* luciferase under the control of the SV-40 promoter with polyA signal including pRF, phpRF, pRMF and phpRMF were previously described (14) and were received as a gift from Professor Anne Willis. To study the potential IRES element of hnRNP K, 259 nucleotides variant 1 (or variant 3) and variant 2 (224 nucleotides) of human hnRNP K 5′ untranslated region (UTR) were amplified by PCR from the complementary DNA (cDNA) of HEK 293 cells. The mouse hnRNP K 5′ UTR (151 nucleotides) were amplified from cDNA of neuro-2a cells. The purified PCR products (containing two restriction enzyme sites *Spe*I and *Nco*I) containing different fragments of hnRNP K 5′ UTR were used to perform TA cloning (Yeastern Biotech Co. Ltd.) and subcloned from yT&A plasmid into pRF and phpRF bicistronic reporter vectors to produce pRK1F, pRK2F, phpRK1F and phpRK2F reporter constructs. The mouse hnRNP K 5′ UTR was subcloned from yT&A into phpRF bicistronic reporter vectors to produce phpRmKF reporter construct. To establish monocistronic reporter assay, the PCR products (containing two restriction enzyme sites *Hind*III and *Nco*I) of human hnRNP K were subcloned from yT&A into pGL3 promoter (Promega) plasmid to produce pGL3PK1 and pGL3PK2 reporter constructs. To perform biotin pull-down assay, the purified PCR products (containing two restriction enzyme sites *Spe*I and *Nco*I) containing different fragments of human 5′ UTR were cloned into pGEM-T-Easy plasmid (Promega) to produce K1-pGEM-T-Easy (contains 259 nucleotides of hnRNPK 5′ UTR), K2-pGEM-T-Easy (contains 224 nucleotides of hnRNPK 5′ UTR) and nucelolin-pGEM-T-easy (contains 141 nucleotides of hnRNPK 5′ UTR) plasmids. To generate expression plasmid for hnRNP K, the purified PCR product containing coding sequence of human hnRNP K was used to perform TA cloning and then subcloned into pcDNA3-HA plasmid to produce HA-hnRNP K plasmid.

The siRNA against hnRNP K was purchased from Ambion (Silencer Pre-designed siRNA) and the sequence was as follows: sense, 5′ GCGCAUAUUGAGUAUCAGU 3′; antisense, 5′ ACUGAUACUCAAUAUGCGC 3′. The siRNA of negative control was purchased from Sigma and the sequence was as follows: sense, 5′ GAUCAUACGUGCGAUCAGA 3′; antisense, 5′ UCUGAUCGCACGUAUGAUC 3′.

Transfections were performed using TurboFect transfection reagent (Thermo Scientific), as per manufacture's recommendation. Twenty-four hours after transfection, the protein expression level of hnRNP K was determined by western blotting analysis. Firefly and Renilla luciferase activity was measured by a luminometer (Turner Designs) using a Dual Luciferase Reporter kit (Promega). Transfection efficiency was corrected by normalizing the data to the corresponding Renilla luciferase (phRGTK; Promega) activity for pGL3P, pGL3PK1 or pGL3PK2 construct.

### Antibodies

Mouse monoclonal anti-hnRNP K (D-6) antibody and rabbit polyclonal anti-nucleolin (H-250), normal mouse immunoglobulin G (IgG) and normal rabbit IgG antibodies were purchased from Santa Cruz Biotechnology. The rabbit polyclonal anti-human actin antibodies and rabbit polyclonal anti-glial fibrillary acidic protein (GFAP) antibodies were purchased from Sigma. The mouse monoclonal HA antibody (MMS-101R) was purchased from Covance. The rabbit polyclonal anti-MOR antibodies (GTX10275), rabbit polyclonal anti-NeuN antibodies (GTX37604), mouse monoclonal Myc-tag antibody (9E10) and mouse monoclonal anti-α-tubulin antibody (GTX72360) were purchased from GeneTex. The rabbit polyclonal anti-Sp1 antibodies were purchased from Millipore. The Alexa-488 goat anti-rabbit IgG, Alexa-488 goat anti-mouse IgG, Alexa-568 goat anti-rabbit IgG and Alexa-568 goat anti-mouse IgG antibodies were purchased from Invitrogen.

### Subcellular fractionation of HEK-MOR cells

HEK-293 cells were rinsed with phosphate-buffered saline (PBS) and lysed with lysis buffer (10 mM HEPES, pH 8.0, 40 mM KCl, 3 mM MgCl_2_, 5% glycerol, 2 mM DTT, 0.5% Nonidet P-40) for 10 min at 4°C. Whole cell lysates were prepared by performing centrifugation (4250 *g* for 5 min at 4°C), and the supernatant was collected and stored at −80°C. For cytoplasmic and nuclear extracts preparation, cells were fractionated using Subcellular Protein Fractionation Kit for Culture Cells (Thermo Scientific) according to the manufacturer's instructions. Briefly, cells were harvested with trypsin-ethylenediaminetetraacetic acid and then washed with ice-cold PBS. The cytoplasmic and nuclear extracts were extracted by incubating cells with cytoplasmic extraction buffer or nuclear extraction buffer, respectively.

### Protein degradation assay

HEK-293 cells were grown to 80% confluence, followed by addition of cycloheximide (1 mg/ml). Total proteins were harvested and lysed from the cells at different time points using radio-immunoprecipitation assay lysis buffer (150 mM NaCl, 20 mM Tris-Cl pH 7.4, 1% NP-40, 1% Triton X-100, 0.1% sodium dodecyl sulphate (SDS)) supplemented with protease inhibitor cocktail (Roche). The resulting proteins were analyzed by western blotting with hnRNP K and actin antibodies. The protein turnover rate was normalized by the house keeping gene, *actin*.

### Quantitative real-time PCR and reverse transcription

The primers used to perform real-time PCR were listed in Supplementary Table S2. Total RNAs were isolated and reverse transcribed using Superscript III Reverse Transcriptase (Invitrogen) according to manufacturer's instructions. Quantitative real-time PCR were performed using SYBR Green PCR Master mix (Applied Biosystems) and analyzed with ABI PRISM 7900HT sequence detection system (Applied Biosystems).

### ^35^S-Methionine incorporation

At various time points after morphine treatment, HEK-MOR cells with the density of 5 × 10^5^ cells/cm^2^ were washed with PBS and incubated in methionine-free DMEM (Invitrogen), but containing 30 μCi/ml L-^35^S-methionine/cysteine (Perkin Elmer) for 1 h.

### RNA biotinylation and *in vitro* transcription

To synthesize the biotin-labeled 5′ UTR, the plasmids including K1-pGEM-T-Easy, K2-pGEM-T-Easy and nucelolin-pGEM-T-easy were linearized through restriction enzyme digestion using *Nde*I. Three biotin-labeled 5′ UTR were synthesized using the Riboprobe *in vitro* Transcription System (Promega). Following manufacturer's instructions, 1 μg of linearized DNA was incubated with the transcription optimized buffer (100 mM DTT, 2.5 mM γATP, 2.5 mM γGTP, 2.5 mM γUTP, 100 μM γCTP), 10 mM biotin-14 CTP (Invitrogen), 1 unit/μl of recombinant RNase ribonuclease inhibitor and T7 RNA polymerase for 2 h at 37°C. The reaction mixture was then treated with RQ1 DNase (Promega) for 15 min at 37°C and purified using Microspin G-25 columns (GE Healthcare).

### Biotin pull-down assay

The 500 μg cytoplasmic extracts of HEK-293 were incubated with 20 pM biotin-labeled RNA and rotated for 24 h at 4°C. The reaction mixture was incubated with NeutrAvidin agarose resin (Thermo Scientific) for 2 h at 4°C, and then washed three times with wash buffer (10 mM HEPES pH 8.0, 40 mM KCl, 3 mM MgCl_2_, 5% glycerol, 2 mM DTT, 0.5% Nonidet P-40, 1% Tween-20) supplemented with protease inhibitor cocktail (Roche). The RNA-protein complex was analyzed by western blotting with hnRNP K antibody, nucleolin antibodies and actin antibodies.

### RNA-immunoprecipitation assay

The 1 mg cytoplasmic extracts from HEK-293 cells were incubated with 1 μg mouse anti-hnRNP K antibody or rabbit anti-nucleolin antibodies for 24 h at 4°C. The normal mouse IgG and normal rabbit IgG were used as negative controls for immunoprecipitation assay. Then the reaction mixture was incubated with protein A/G agarose beads (GE healthcare) for 2 h at 4°C. Immunoprecipitated complexes were washed three times with cytoplasm lysis buffer, and bound RNAs were extracted by TRIzol reagent (Invitrogen). After synthesis of complementary DNA (cDNA) using reverse transcription (Invitrogen), quantitative real-time PCR were used to detect hnRNP K or nucleolin mRNA levels.

### Immunostaining

C57BL/6 (B6) mice were perfused and fixed with 4% paraformaldehyde in PBS for 2 h at 4°C. The brain section was glued to the chuck of a Vibroslice tissue slicer. Transverse slices of 10 μm thickness were cut and the appropriate slices from control (vehicle) and experimental groups were placed on the same microscope slide and processed identically at the same time in the immunofluorescence staining procedure. HEK-293 cells were washed with cold PBS and fixed at room temperature for 20 min with 4% paraformaldehyde. After washing, slices or cells were incubated with permeabilization buffer (0.4% Triton X-100 and 2% FBS in PBS) for 1 h. Expression of hnRNP K, MOR, NeuN or GFAP was detected using mouse anti-hnRNP K antibody (1:200), rabbit anti-MOR antibodies (1:200), rabbit anti-NeuN antibodies (1:200) or rabbit anti-GFAP antibodies (1:200), respectively, in PBS for 2 h. Cells were washed four times with wash buffer (0.2% Triton X-100 in PBS) and incubated in PBS with Alexa488-conjugated goat anti-mouse antibody (1:200), Alexa488-conjugated goat anti-rabbit antibodies (1:200), Alexa568-conjugated goat anti-mouse antibody (1:200), Alexa568-conjugated goat anti-rabbit antibodies (1:200) and 4',6-diamidino-2-phenylindole (DAPI) for 1 h. The slides were washed three times with PBS and mounted with glycerol. Images were visualized using fluorescence microscopy (Nikon) and acquired at the same gain and exposure time.

### Membrane potential assay

The mouse pituitary AtT-20 cells were transiently transfected with vehicle or a myc-tagged MOR plasmid using electroporation and seeded in 96-well plates. After 24 h, cells were serum-starved for 3 h to detect potassium conductance changes using a fluorometric imaging plate reader (FLIPR) membrane potential assay according to the manufacturer's instructions (Molecular Devices). Briefly, serum-starved cells were treated with blue membrane potential dye for 0.5 h at 25°C. The fluorescence signal (excitation: 485 nm, emission: 525 nm) were monitored every 1.52 s interval, up to 150 s after morphine injection on the FlexStation 3 benchtop multi-mode microplate reader (Molecular Devices).

### Spinal cord surgery and tail-flick test

Male wild-type (WT) B6 mice (25–30 g) and MOR-KO mice (15) (kindly provided by Dr. Pao-Luh Tao, National Health Research Institutes, Taiwan) were kept in a temperature-controlled animal room with a 12-h light/dark cycle. The protocol has been approved by the Institutional Animal Care and Use Committee of the National Health Research Institutes, Taiwan. Animal experiments were carried out in accordance with the Policies on the Use of Animals in Neuroscience Research and the ethical guidelines for investigations of experimental pain in conscious animals, International Association for the Study of Pain.

To reduce hnRNP K expression in the spinal cord, hnRNP K siRNA was dissolved in artificial cerebrospinal fluid and injected intrathecally into adult mice. Briefly, vehicle, 1 nM of hnRNP K siRNA or siRNA of negative control was injected into the spinal cord using Micro-Renathane implantation tubing (Braintree Scientific) inserted in the T7–T8 intervertebral disc. After injection, the NEPA21 electroporator gene transfection system (Nepa Gene) was used to deliver siRNA into cells through needle electrodes inserted between the T9 and L2 vertebrae (16). The poring pulse conditions for electroporation were as follows: 150 V, pulse length of 5 ms, inter-pulse intervals of 50 ms and a 10% decay rate with plus polarity. The transfer pulse conditions were as follows: 20 V, 50-ms pulse length, 50-ms pulse interval and a 40% decay rate with plus and minus polarities. Three days after surgery, a Tail-Flick Analgesia Meter (Columbia Instruments) was used to measure the tail-flick latencies of mice. The cut-off time for each measurement was 10 s to avoid tissue damage. Basal latencies were recorded before morphine administration and test latencies were recorded 0.5 and 1 h after an intravenous injection of morphine (10 mg/kg). A time–response curve was calculated for antinociceptive effects (test latency − basal latency) occurring during 0–60 min. We confirmed that mice received vehicle injection had no significant tissue injury.

### Quantification of cell number

The total hnRNP K-positive cell number in each region was estimated according to Cavalieri principle (17,18):

\begin{equation*} N({\rm cell},{\rm reg}) = \Sigma \;Q^ - /\Sigma \;v({\rm dis})*V({\rm reg}) \end{equation*}

*N*(cell, reg) is the estimated cell number in each region, Σ *Q*^−^ is the number of cells counted in all dissectors in a region, Σ *v*(dis) is the volume of dissectors equal to the area of the counting frame and *V*(reg) is the reference volume of each region. Five serial 10-μm-thick sections spaced 50 μm apart were counted manually under a fluorescence microscopy at 200x magnification.

### Statistical analysis

The data were collected from experiments performed in triplicate, and were expressed as the mean ± standard deviation (SD). Comparisons among multiple groups were performed using a one-way ANOVA with appropriate *post hoc* tests, whereas comparisons between two groups were performed using Student's *t*-test (StatView 5.01; SAS Institute). A *P*-value of *P* ≤ 0.05 was considered statistically significant.

## RESULTS

### Morphine up-regulates hnRNP K expression in the CNS

**Figure 1.**
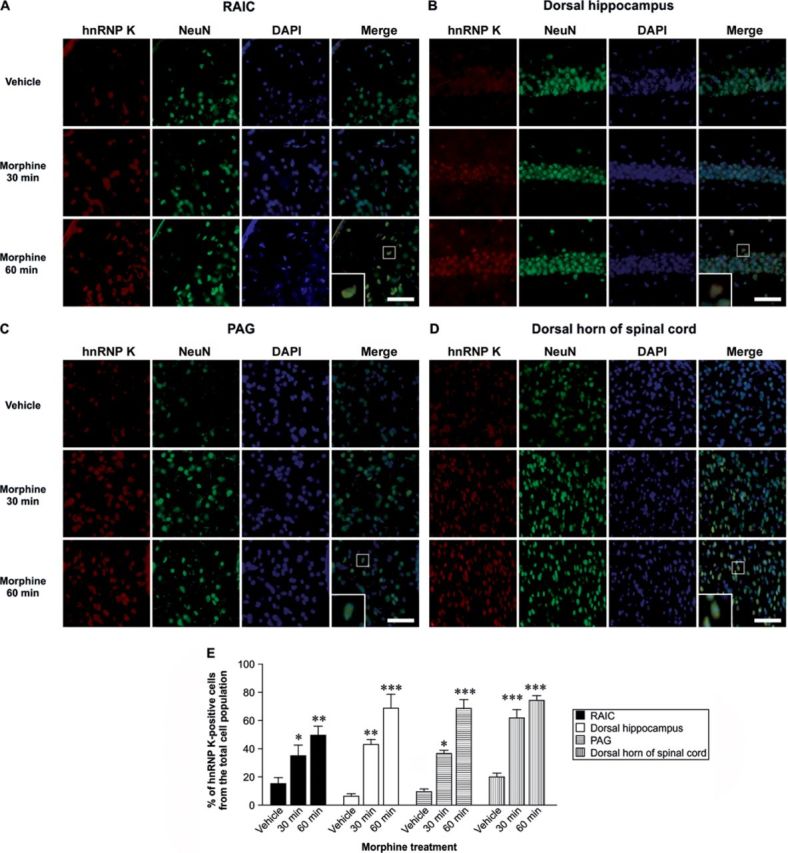
Morphine-induced hnRNP K protein expression in mouse CNS. Morphine increased hnRNP K protein expression in the RAIC (A), dorsal hippocampus (B), PAG (C) and dorsal horn of spinal cord (D) of B6 mice. The B6 mice were injected with vehicle or morphine (10 mg/kg, i.v.) and sacrificed at the indicated time points (30 and 60 min). The expression levels of hnRNP K (red) and NeuN (green, a neuronal marker) were visualized using immunofluorescence assay. DAPI (blue) is used as a nuclear marker for orientation. Scale bars, 50 μm. (E) Quantification of hnRNP K-positive cells in the total cell population was presented as the mean ± SD. Statistical analysis was carried out using the one-way ANOVA with appropriate *post hoc* tests. *P < 0.05, **P < 0.01, ***P < 0.001 versus vehicle control group.

MOR is mostly present in the CNS and its activation induced by morphine produces strong analgesia (19). To determine the effects of morphine on hnRNP K protein expression, mice were injected intravenously (i.v.) with morphine (10 mg/kg) and hnRNP K expression levels (red) were visualized using immunofluorescent staining. Compared to the vehicle group, morphine treatment resulted in a time-dependent increase in hnRNP K expression in MOR-rich region, such as rostral agranular insular cortex (RAIC) (Figure 1A; Figure 1E, *F*_2,12_ = 7.6, *P* < 0.01), dorsal hippocampus (Figure 1B; Figure 1E, *F*_2,12_ = 23.59, *P* < 0.001), periaqueductal gray (PAG) (Figure 1C; Figure 1E, *F*_2,12_ = 53.61, *P* < 0.001) and dorsal horn of spinal cord (Figure 1D; Figure 1E, *F*_2,12_ = 39.51, *P* < 0.001) of mice. A Newman–Keuls *post hoc* comparison revealed significant differences between the vehicle control and morphine-treated groups (RAIC: 30 min, *P* < 0.05; 60 min, *P* < 0.01; dorsal hippocampus: 30 min, *P* < 0.01; 60 min, *P* < 0.001; PAG: 30 min, *P* < 0.05; 60 min, *P* < 0.001; dorsal horn of spinal cord: 30 min and 60 min, *P* < 0.001). It is noted that hnRNP K did not co-localize with GFAP, an astrocyte marker (Supplementary Figure S1), indicating that morphine-induced hnRNP K expression is neuron-specific. Thus, our results indicate that morphine-induced hnRNP K expression in CNS regions with a major distribution of MOR.

### Morphine increases hnRNP K expression in HEK-MOR cells and rat primary cortical neurons

**Figure 2.**
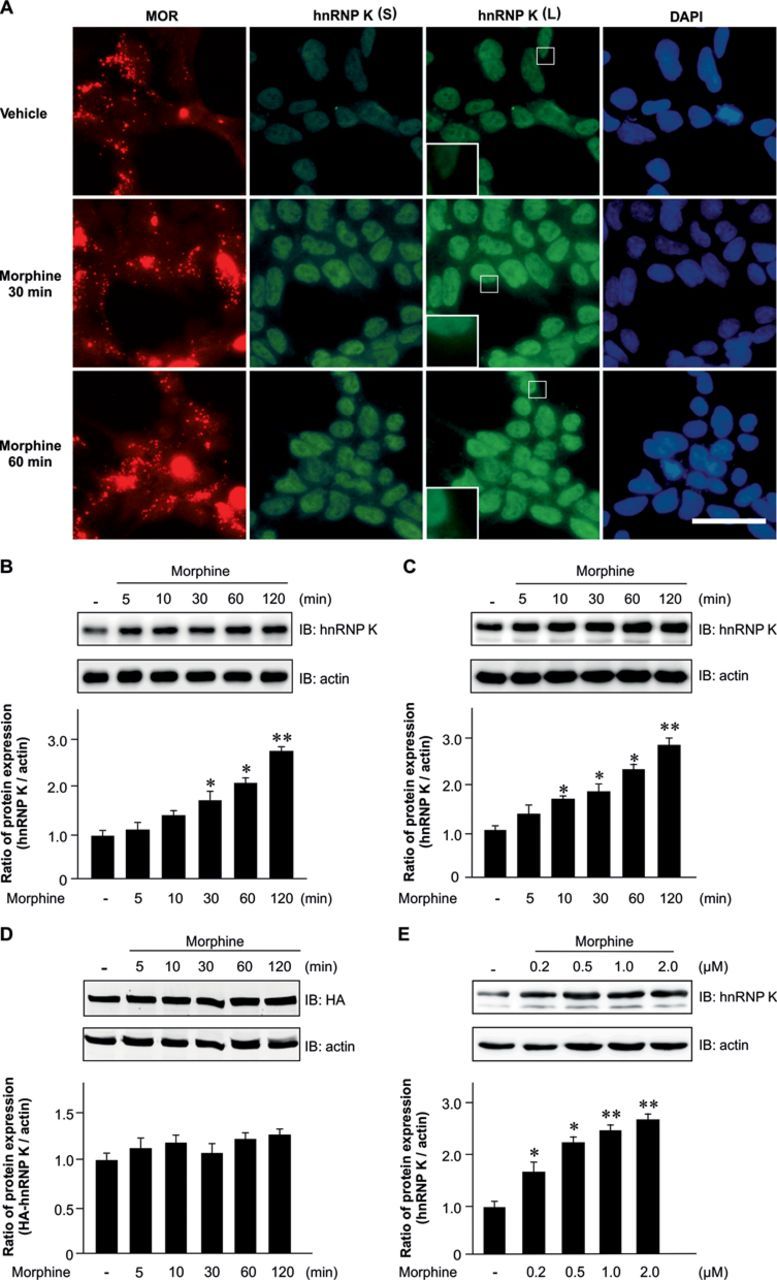
Morphine-induced hnRNP K protein expression in HEK-MOR cells and rat primary cortical neurons. (A) Morphine induced both nuclear and cytoplasmic hnRNP K expression in HEK-MOR cells. HEK-MOR cells were treated with vehicle or morphine (1 μM) at the indicated time points (30 and 60 min). hnRNP K (green) and MOR (red) were visualized by fluorescence microscopy. DAPI (blue) is used as a nuclear marker. Scale bars, 50 μm. (B) and (C) Morphine up-regulated hnRNP K protein expression in HEK-MOR cells (B) and rat primary cortical neurons (C). HEK-MOR cells or rat primary cortical neurons were treated with vehicle or morphine (1 μM) at the indicated time points (5, 10, 30, 60 and 120 min), and hnRNP K protein expression was analyzed by western blotting. (D) Morphine failed to induce HA-hnRNP K up-regulation in HEK-MOR cells. HA-hnRNP K plasmid was transiently transfected into HEK-MOR cells and then treated with morphine (1 μM) at the indicated time points. Whole cell lysates were analyzed by western blotting. (E) Morphine elevated protein expression of hnRNP K in a dose-dependent manner. HEK-MOR cells were treated with vehicle or various concentration of morphine (0.2, 0.5, 1 or 2 μM) and whole cell lysates were analyzed by western blotting. All experiments were carried out independently and at least in triplicate. Protein expression was quantified using densitometry (lower panels in B, C, D and E). The values indicate the mean ± SD. Statistical analysis was carried out using one-way ANOVA with appropriate *post hoc* tests. *P < 0.05, **P < 0.01 versus vehicle control group. S, short exposure; L, long exposure.

To study hnRNP K expression upon morphine stimulation, HEK-MOR cells were treated with morphine and hnRNP K expression were determined by using immunofluorescent staining and western blotting analysis. Results show that morphine increased both nuclear (Figure 2A, short exposure; Supplementary Figure S2, right panel) and cytoplasmic (Figure 2A, long exposure; Supplementary Figure S2, left panel) hnRNP K expression. Furthermore, morphine increases hnRNP K levels in a time-dependent (Figure 2B; *F*_5,12_ = 6.75, *P* < 0.01) and dose-dependent manner (Figure 2E; *F*_4,10_ = 11.35, *P* < 0.001). A Newman–Keuls *post hoc* comparison revealed significant differences between the control and morphine-treated groups (Figure 2B; 30 and 60 min, *P* < 0.05; 120 min, *P* < 0.01. Figure 2E; 0.2 and 0.5 μM, *P* < 0.05; 1.0 and 2.0 μM, *P* < 0.01). In addition, morphine up-regulated hnRNP K expression in rat primary cortical neurons (Figure 2C; *F*_5,12_ = 56.18, *P* < 0.001). A Newman–Keuls *post hoc* comparison revealed significant differences between the control and the morphine-treated groups (10, 30 and 60 min, *P* < 0.05; 120 min, *P* < 0.01). However, the effect of morphine on hnRNP K expression was not observed in HEK-MOR cells overexpressing HA-tagged hnRNP K without UTRs of its transcript (Figure 2D; *F*_5,12_ = 1.05, *P* > 0.05), suggesting that its UTRs may be involved in morphine-induced up-regulation of hnRNP K. Thus, our results demonstrated that morphine increased hnRNP K protein levels in HEK-MOR cells and rat primary cortical neurons.

### Morphine activates translational machinery to promote hnRNP K synthesis in HEK-MOR cells

**Figure 3.**
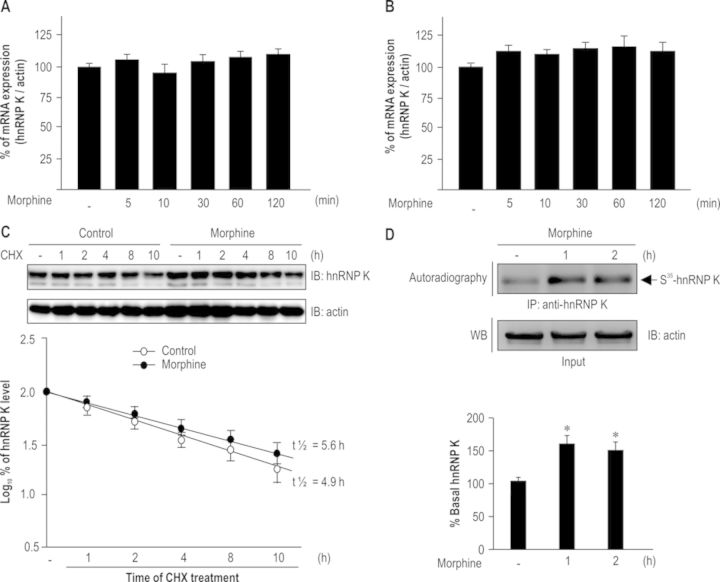
Morphine elevated hnRNP K expression through translational control. Morphine exerted no effects on hnRNP K mRNA expression in HEK-MOR cells (A) or rat primary cortical neurons (B). HEK-MOR cells or rat primary cortical neurons were treated with vehicle or morphine (1 μM) at the indicated time points (5, 10, 30, 60 and 120 min). hnRNP K mRNA expression levels were detected using quantitative RT-PCR and normalized with β-actin mRNA. (C) Morphine did not alter protein degradation rate of hnRNP K. HEK-MOR cells were pre-treated with vehicle or morphine (1 μM) for 2 h. The cells were then treated with a protein synthesis inhibitor, CHX, at different time points (1, 2, 4, 8 and 10 h). hnRNP K expression was detected using western blotting and quantified using densitometry. (D) Morphine treatment enhanced newly synthesized hnRNP K protein. HEK-MOR cells were labeled with ^35^S-methionine/cysteine flowing morphine treatment. The whole-cell lysates were immunoprecipitated with anti-hnRNP K antibody and fractionated by SDS-polyacrylamide gel electrophoresis followed by autoradiography. Radiolabeled newly synthesized hnRNP K protein was quantified using densitometry. *P < 0.05 versus vehicle control group. All experiments were carried out independently and at least in triplicate. The values indicate the mean ± SD. Statistical analysis was carried out using one-way ANOVA with appropriate *post hoc* tests.

To identify the underlying mechanism of morphine to augment hnRNP K expression, we evaluated whether morphine altered transcriptional activity, protein stability or protein synthesis of hnRNP K. First, we observed morphine could not alter hnRNP K mRNA levels in HEK-MOR cells (Figure 3A; *F*_5,12_ = 2.21, *P* > 0.05) and rat primary cortical neurons (Figure 3B; *F*_5,12_ = 2.98, *P* > 0.05). Second, to determine whether the regulation of protein stability contributed to morphine-induced hnRNP K up-regulation, HEK-MOR cells were treated with vehicle or morphine for 2 h, followed by cycloheximide (CHX) to block *de novo* protein synthesis. Figure 3C shows a slight but not significant increase in the protein half-life of morphine pre-treated group (4.9 h) as compared to vehicle control group (5.6 h), indicating that the protein stability of hnRNP K was not affected by morphine. Third, we utilized ^35^S-methionine incorporation assays to determine whether morphine increased newly synthesized hnRNP K in HEK-MOR cells. Figure 3D reveals that newly synthesized hnRNP K was significantly increased after morphine treatment (*F*_2,6_ = 9.693, *P* < 0.05). A Newman–Keuls *post hoc* comparison revealed significant differences between the control and morphine-treated groups (1 and 2 h, *P* < 0.05). These results demonstrate that morphine up-regulated hnRNP K expression through translational control.

### Conserved sequence of hnRNP K 5′ UTR facilitates translation

**Figure 4.**
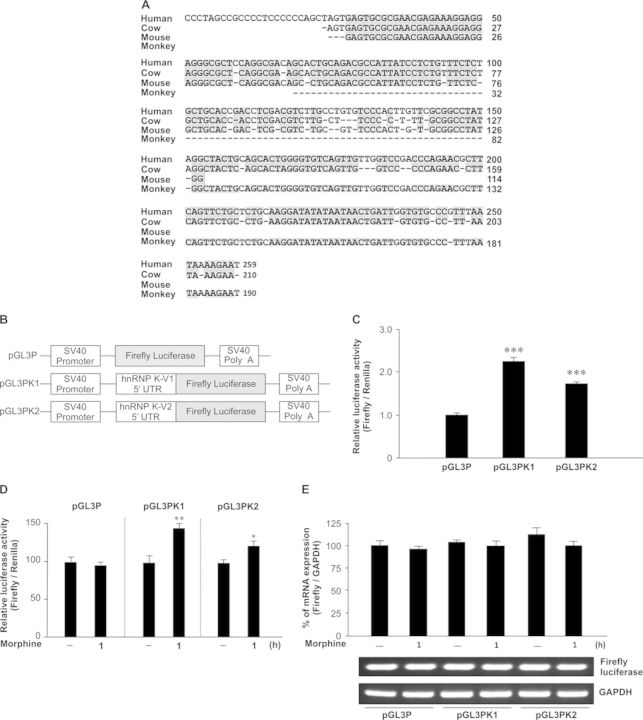
The 5′ UTR of hnRNP K contains a conserved sequence across different species. (A) Multiple sequence alignment of hnRNP K 5′ UTR from different species. The diagram illustrates the conserved sequences of hnRNP K 5′ UTR across different species including human, cow, monkey and mouse. The strictly conserved nucleotides were colored on a gray background. (B) The diagram of pGL3P (empty vector), pGL3PK1 and pGL3PK2 Firefly luciferase reporter constructs. (C) The reporter plasmids including pGL3P, pGL3PK1 or pGL3PK2 were transiently co-transfected with phRGTK Renilla luciferase reporter plasmid into HEK-MOR cells. The relative luciferase activities were detected using a reporter assay and expressed as the ratio of Firefly to Renilla luciferase activity. ***P < 0.001 versus pGL3P group, one-way ANOVA with appropriate *post hoc* tests. (D) HEK-MOR cells transiently co-transfected with reporter plasmids described in (C) were treated with vehicle or morphine (1 μM) for 1 h and subjected to a reporter assay. *P < 0.05, **P < 0.01 versus corresponding vehicle plasmid group, Student's *t*-test. (E) HEK-MOR cells transiently transfected with pGL3P, pGL3PK1 or pGL3PK2 plasmids were treated with vehicle or morphine (1 μM) for 1 h and the mRNA levels of Firefly luciferase were analyzed by quantitative real-time RT-PCR and normalized with GAPDH mRNA. All experiments were carried out independently and at least in triplicate. The values indicate the mean ± SD.

The 5′ UTR of mRNA plays a crucial role in regulating internal translation in stressful conditions (12). The nucleotide sequences of hnRNP K from GenBank were collected to perform multiple sequence alignment for different species including human (NM_002140.3), cow (NM_001034562.1), mouse (NM_002579.2) and monkey (NM_001266946.1). The results revealed that four species shared sequence similarity and contain conserved sequences of hnRNP K 5′ UTR across different species (Figure 4A). Therefore, evolutionary conservation of the hnRNP K 5′ UTR sequence exists among different species, suggesting a potential regulatory function. Three isoforms of human hnRNP K mRNA including transcript variant 1, 2 and 3 have been identified. The 5′ UTR of variant 1 and 3 contain the same 259 nucleotides and the 5′ UTR of variant 2 contains 224 nucleotides. To examine whether human hnRNP K 5′ UTR can regulate its translation, the 5′ UTRs of variant 1 and 2 were cloned, respectively, into pGL3 promoter to produce monocistronic reporter plasmids pGL3PK1 and pGL3PK2 (Figure 4B). All groups including reporter plasmid pGL3P (empty vector), pGL3PK1 or pGL3PK2 were transiently co-transfected with phRGTK Renilla luciferase reporter plasmid to normalize for transfection efficiency differences. Their relative luciferase activities were detected using a reporter assay. We found the translation efficiency of both pGL3PK1 and pGL3PK2 were higher than that of pGL3P (Figure 4C; *F*_2,6_ = 66.54, *P* < 0.001). A Newman–Keuls *post hoc* comparison revealed significant differences (pGL3PK1: *P* < 0.001; pGL3PK2: *P* < 0.001). Furthermore, morphine could enhance hnRNP K 5′ UTR-mediated increase in luciferase activity. A Student's *t*-test revealed significant differences between the control and morphine-treated groups (Figure 4D; pGL3PK1: *P* < 0.01; pGL3PK2: *P* < 0.05). Finally, we observed similar mRNA levels among pGL3P, pGL3PK1 and pGL3PK2 groups treated with vehicle or morphine (Figure 4E; *F*_5,12_ = 2.39, *P* > 0.05) to rule out the possibility that hnRNP K 5′ UTR could act as transcriptional enhancer and be regulated by morphine. Here, we revealed that the isoforms of *hnRNP K* mRNA contain conserved 5′ UTR and plays a functional role in protein synthesis via translational control.

### hnRNP K 5′ UTR demonstrates IRES activity

**Figure 5.**
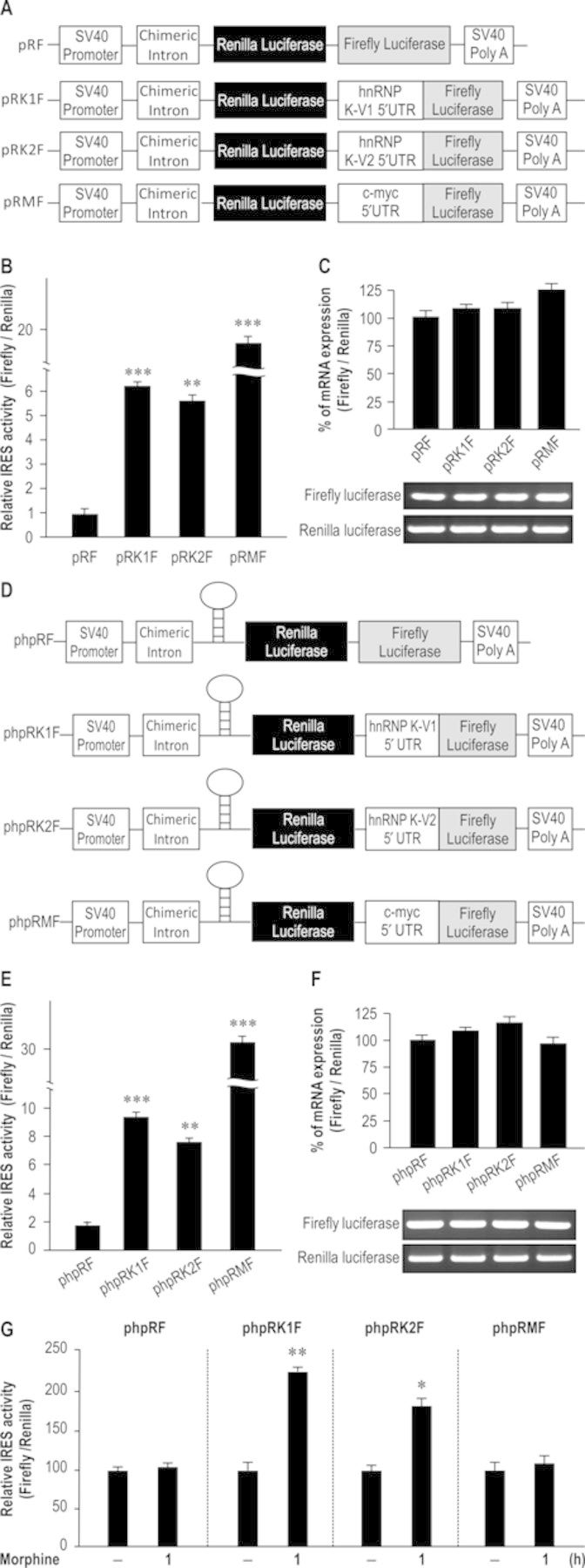
The 5′ UTR of human hnRNP K possesses IRES activity that was regulated by morphine. (A) The diagram of pRF, pRK1F, pRK2F and pRMF bicistronic reporter constructs. The 5′ UTR of human *hnRNP K* mRNA was cloned into bicistronic reporter plasmid to generate pRK1F and pRK2F reporter constructs. The pRMF reporter construct, a well-identified IRES-containing proto-oncogene, was used as a positive control. (B) The 5′ UTR of human *hnRNP K* mRNA shows IRES activity. The indicated four reporter plasmids were transiently transfected into HEK-MOR cells. The ratio of (Firefly/Renilla) was analyzed by reporter assay. **P < 0.01, ***P < 0.001 versus pRF group, one-way ANOVA with appropriate *post hoc* tests. (C) pRF, pRK1F, pRK2F and pRMF were transiently transfected into HEK-MOR cells. The mRNA ratio (Firefly/Renilla) was calculated from data obtained with quantitative real-time RT-PCR. (D) The diagram of phpRF, phpRK1F, phpRK2F and phpRMF bicistronic reporter constructs. The 5′ UTR of human *hnRNP K* mRNA was cloned to generate the phpRK1F and phpRK2F reporter constructs. The stable hairpin structure has been reported to inhibit cap-dependent translation. (E) The 5′ UTR of human *hnRNP K* mRNA shows IRES activity. The indicated four reporter plasmids were transiently transfected into HEK-MOR cells. The ratio of (Firefly/Renilla) was analyzed by reporter assay. **P < 0.01, ***P < 0.001 versus phpRF group, one-way ANOVA with appropriate *post hoc* tests. (F) After transfection, the mRNA ratio (Firefly/Renilla) of four constructs was analyzed by quantitative real-time RT-PCR. (G) Morphine enhanced IRES activity of hnRNP K. The reporter plasmids were transiently transfected into HEK-MOR cells, and treated with vehicle or morphine (1 μM) and harvested at the indicated time point (1.0 h). The ratio of Firefly/Renilla was analyzed by reporter assay. *P < 0.05, **P < 0.01 versus vehicle control group, Student's *t*-test. All experiments were carried out independently and at least in triplicate. The values indicate the mean ± SD.

Previous reports indicate that bicistronic reporter constructs, such as pRF, pRMF, phpRF and phpRMF, can be used to study cellular IRES elements (14). To investigate whether *hnRNP K* mRNA shows IRES activity, hnRNP K 5′ UTR of variant 1 and 2 were cloned, respectively, to the middle region between two coding regions of Renilla luciferase and Firefly luciferase to produce bicistronic reporter construct pRK1F (variant 1) and pRK2F (variant 2). The pRMF construct contains c-myc IRES activity as the positive control (Figure 5A). The four reporter plasmids were transiently transfected into HEK-MOR cells and luciferase activity was detected using a reporter assay. Figure 5B shows that, compared with the empty vector (pRF), both hnRNP K 5′ UTRs caused approximate 6-fold increase in reporter activity (*F*_3,8_ = 110.5, *P* < 0.001). A Newman–Keuls *post hoc* comparison revealed significant differences between the control and experimental groups (pRF versus pRK1F: *P* < 0.001; pRF versus pRK2F: *P* < 0.01; pRF versus pRMF: *P* < 0.001). To determine whether protein translation of Renilla luciferase and Firefly luciferase were produced from the same RNA transcript, the percentage of mRNA ratio (Firefly/Renilla) of the cells were determined by using quantitative reverse transcriptase-PCR (RT-PCR). No significant differences were observed in the mRNA ratios among these groups (Figure 5C; *F*_3,8_ = 2.05, *P* > 0.05). These results suggest the translation efficiency was increased after linkage of hnRNP K 5′ UTR to reporter genes.

Next, to isolate IRES-dependent translation, we used bicistronic reporter plasmids with a hairpin structure (Figure 5D), which inhibits cap-dependent translation via repression of ribosome screening (20). Compared with phpRF (empty vector), phpRK1F (variant 1) and phpRK2F (variant 2) showed ∼8- to 10-fold increase in translation efficiency (Figure 5E; *F*_3,8_ = 49.71, *P* < 0.001). These significant differences were observed in comparison with the control group using a Newman–Keuls *post hoc* test (phpRF versus phpRK1F: *P* < 0.001; phpRF versus phpRK2F: *P* < 0.01; phpRF versus phpRMF: *P* < 0.001). Similar control experiments carried out in Figure 5C were performed here and no significant differences among these groups were observed (Figure 5F; *F*_3,8_ = 1.78, *P* > 0.05). Based on the results described above, the 5′ UTR of *hnRNP K* mRNA contains significant IRES activity to modulate translational control. Finally, we identified that morphine treatment led to a significant increase in the IRES activity of hnRNP K 5′ UTR. A Student's *t*-test revealed significant differences between the control and morphine-treated groups (Figure 5G; phpRK1F: *P* < 0.01; phpRK2F: *P* < 0.05). These results demonstrate hnRNP K 5′ UTR contains IRES activity, and can be activated by morphine.

### hnRNP K protein interacts with its 5′ UTR of mRNA

**Figure 6.**
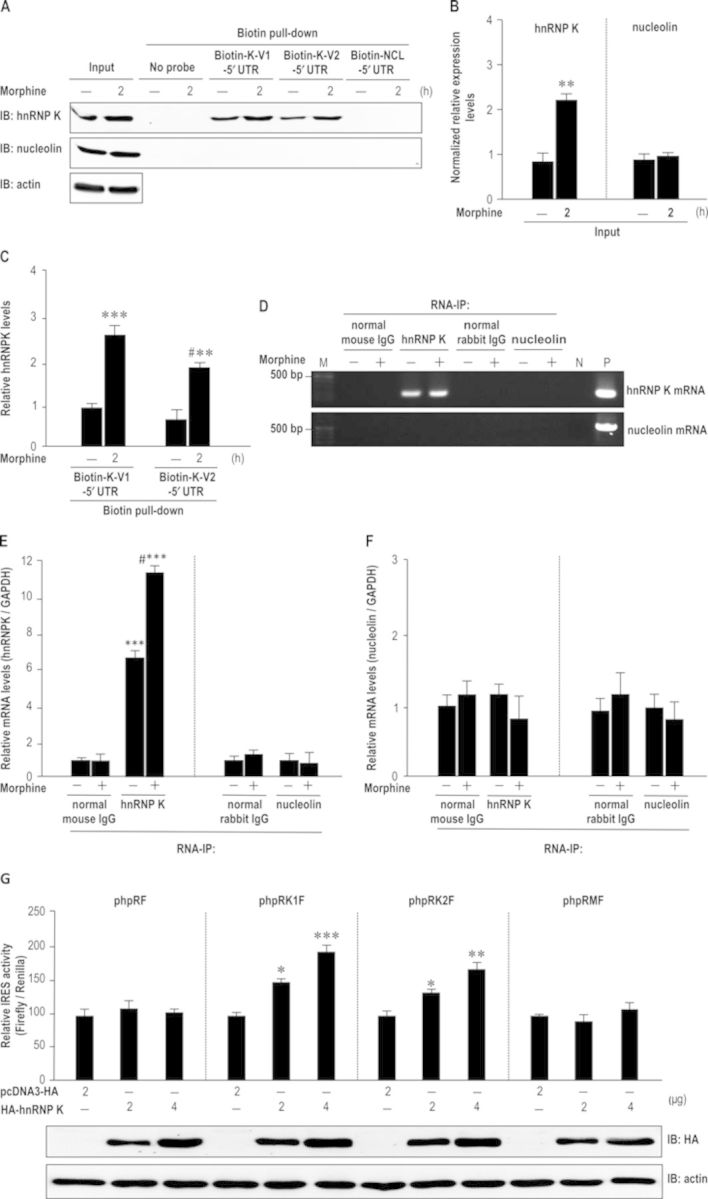
Morphine-enhanced hnRNP K bound to its 5′ UTR. (A) The RNA-protein interaction between hnRNPK and biotinylated hnRNP K 5′ UTR was regulated by morphine *in vitro*. HEK-MOR cells were treated with vehicle or morphine (1 μM) for 2 h. Cytosolic fractions were extracted and incubated with biotin-labeled hnRNP K 5′ UTR (Biotin-K-V1–5′ UTR or Biotin-K-V2–5′ UTR) or biotin-labeled nucleolin 5′ UTR (Biotin-NCL-5′ UTR). The protein-RNA interaction was detected using biotin pull-down analysis with a specific antibody against hnRNP K or nucleolin. (B) Quantification of protein expression was measured using densitometry. The results of western blotting from (A) revealed morphine increased hnRNP K expression. **P < 0.01 versus vehicle group, Student's *t*-test. (C) Biotin pull-down analysis from (A) revealed morphine increased the association between hnRNP K protein and its mRNA *in vitro*. **P < 0.01, ***P < 0.001 versus Biotin-K-V1–5′ UTR group. ^#^*P* < 0.05 versus Biotin-K-V2–5′ UTR group, one-way ANOVA with appropriate *post hoc* tests. (D) The protein-RNA interaction between hnRNP K protein and mRNA was regulated by morphine *in vivo*. The cytosolic fractions were extracted and immunoprecipitated with normal mouse IgG, mouse anti-hnRNP K antibody, normal rabbit IgG or rabbit anti-nucleolin antibodies, respectively. The associated hnRNP K/mRNA complex was extracted and analyzed by RT-PCR. The control group was immunized with normal mouse IgG or normal rabbit IgG. N, non-template control; P, positive control; M, DNA marker. (E) The association between hnRNP K or nucleolin protein and *hnRNP K* mRNA was measured using quantitative RT-PCR from (D). ***P < 0.001 versus normal mouse IgG group. ^#^*P* < 0.05 versus anti-hnRNP K antibody group, one-way ANOVA with appropriate *post hoc* tests. (F) The association between hnRNP K or nucleolin and *nucleolin* mRNA was measured using quantitative RT-PCR from (D). (G) Up-regulation of hnRNP K increases IRES-mediated hnRNP K translation. The reporter plasmids and the overexpression vectors were transiently co-transfected into HEK-MOR cells and the ratio of Firefly/Renilla was analyzed by reporter assay. *P < 0.05, **P < 0.01, ***P < 0.001 versus pcDNA3-HA group, one-way ANOVA with appropriate *post hoc* tests. All experiments were carried out independently and at least in triplicate. The values indicate the mean ± SD.

Many IRES trans-acting factors (ITAFs) regulate cap-independent translation by binding to IRES sequences of targeted gene (21). The three transcript variants of human hnRNP K 5′ UTR contain different GC content including 55.6% of variant 1 or 3 and 60.1% of variant 2, which presents a more stable structure and putative binding sites for poly (rC) binding proteins. To determine whether hnRNP K protein could be a morphine-regulated ITAF binding to its mRNA, a biotin pull-down assay was conducted to detect the interaction between hnRNP K protein and its 5′ UTR *in vitro* by using the cytosolic fraction of HEK-MOR cells. First, hnRNP K, rather than nucleolin (an RBP), was significantly increased 2 h after morphine treatment (Figure 6A; Figure 6B; *P* < 0.01, Student's *t*-test). Moreover, results from biotin pull-down assay revealed that hnRNP K protein interacted with biotinylated hnRNP K 5′ UTR of variant 1 (Biotin-K-V1–5′ UTR) and 2 (Biotin-K-V2–5′ UTR) and the interaction was significantly increased by morphine (Figure 6A; Figure 6C; *F*_3,8_ = 33.72, *P* < 0.001). A Newman–Keuls *post hoc* comparison revealed significant differences between the vehicle control and morphine-treated groups (Biotin-K-V1–5′ UTR: *P* < 0.001; Biotin-K-V2–5′ UTR: *P* < 0.05). Biotin-labeled nucleolin 5′ UTR (Biotin-NCL-5′ UTR) was conducted as a control experiment. In addition, we performed RNA-immunoprecipitation in HEK-MOR cells to detect hnRNP K protein-mRNA interaction *in vivo* and found that the complexes precipitated by mouse hnRNP K antibody, but not by rabbit nucleolin antibodies, contained *hnRNP K* mRNA. The interaction between hnRNP K protein and its mRNA was increased by morphine (Figure 6D; Figure 6E, left panel; *F*_3,8_ = 176.6, *P* < 0.001). These significant differences were observed in comparison with the normal mouse IgG group using a Newman–Keuls *post hoc* test (normal mouse IgG versus hnRNP K: *P* < 0.001; normal mouse IgG versus hnRNP K plus morphine: *P* < 0.001). It is worth noting that no nucleolin protein-*nucleolin* mRNA or nucleolin protein-*hnRNP K* mRNA interaction was observed in biotin pull-down analysis (Figure 6A) and RNA-immunoprecipitation assay (Figure 6D; Figure 6E, right panel; *F*_3,8_ = 0.26, *P* > 0.05; Figure 6F, left panel; *F*_2,12_ = 2.89, *P* > 0.05; Figure 6F, right panel; *F*_2,12_ = 4.04, *P* > 0.05), indicating the specificity of hnRNP K protein-*hnRNP K* mRNA interaction. Thus, our results show that hnRNP K binds to its mRNA *in vitro* and *in vivo*. To further demonstrate the IRES activity of hnRNP K 5′ UTR was regulated by hnRNP K protein, the bicistronic reporter constructs (phpRF, phpRK1F, phpRK2F, phpRMF) and the overexpression vectors (HA-hnRNP K or empty vector pcDNA3.1-HA) were transiently co-transfected into HEK-MOR cells. The results indicate that up-regulation of HA-hnRNP K increased the IRES activity of hnRNP K (Figure 6G, upper panel; phpRF: *F*_2,6_ = 1.65, *P* > 0.05; phpRK1F: *F*_2,6_ = 25.35, *P* < 0.01; phpRK2F: *F*_2,6_ = 12.9, *P* < 0.01; phpRMF: *F*_2,6_ = 1.55, *P* > 0.05). A Newman–Keuls *post hoc* comparison revealed significant differences between the pcDNA3.1-HA control group and the HA-hnRNP K overexpression group (phpRK1F: 2 μg, *P* < 0.05; 4 μg, *P* < 0.001; phpRK2F: 2 μg, *P* < 0.05; 4 μg, *P* < 0.01). The efficiency of hnRNP K overexpression was verified by western blotting (Figure 6G, lower panel). These findings reveal that hnRNP K plays a functional role as an ITAF to regulate the IRES activity of its 5′ UTR. Our results suggest that hnRNP K protein can form a complex with 5′ UTR of its transcript, and morphine stimulation increases hnRNP K protein-mRNA interaction to regulate IRES-mediated hnRNP K translation.

### Opioid antagonist naloxone reverses morphine-induced hnRNP K protein accumulation in the CNS of mice

**Figure 7.**
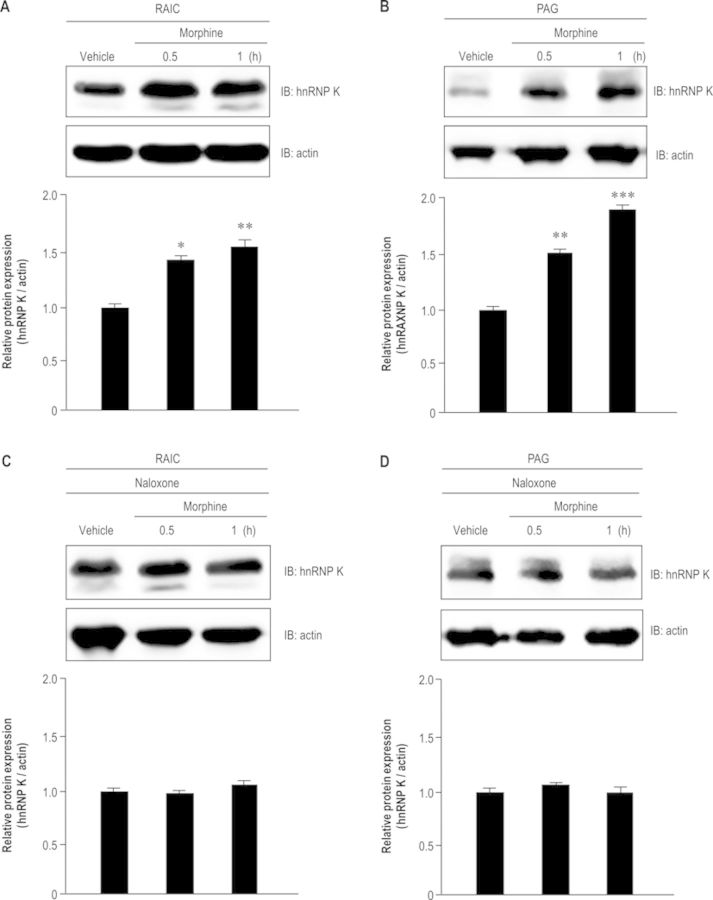
Morphine-induced hnRNP K protein accumulation was reversed by opioid antagonist in CNS of B6 mice. Morphine-stimulated hnRNP K protein accumulation in the RAIC (A) or PAG (B) from mice. The mice were injected with morphine (10 mg/kg, i.v.) and sacrificed at the indicated time points (0.5 and 1 h). The protein expression levels of hnRNP K in the RAIC or PAG were detected using western blotting (A or B, upper panel). Quantification of protein expression was measured using densitometry (A or B, lower panel). Naloxone reverses morphine-stimulated hnRNP K protein accumulation in the RAIC (C) or PAG (D) from mice. The mice were injected with naloxone (10 mg/kg, s.c.) 5 min before vehicle or morphine injection (10 mg/kg, i.v.), and sacrificed at the indicated time points (0.5 and 1 h). The protein expression levels of hnRNP K in the RAIC or PAG were detected using western blotting (C or D, upper panel). Quantification of protein expression was measured using densitometry (C or D, lower panel). All experiments were carried out independently and at least in triplicate. The values indicate the mean ± SD. Statistical analysis was carried out using one-way ANOVA with appropriate *post hoc* tests. *P < 0.05, **P < 0.01, ***P < 0.001 versus vehicle group.

To verify the protein expression levels of hnRNP K under morphine treatment in a mice model, mice were injected with vehicle or morphine (10 mg/kg, i.v.) and the protein levels of hnRNP K in the RAIC and PAG were detected using western blotting. Figure 7A indicates that morphine up-regulated hnRNP K protein expression in the RAIC (*F*_2,6_ = 24.12, *P* < 0.01). A Newman–Keuls *post hoc* comparison revealed significant differences between the vehicle and morphine-treated group (0.5 h, *P* < 0.05; 1 h, *P* < 0.01). Similar results were observed in PAG (Figure 7B; *F*_2,6_ = 16.22, *P* < 0.001). A Newman–Keuls *post hoc* comparison revealed significant differences between the vehicle and morphine-treated group (0.5 h, *P* < 0.01; 1 h, *P* < 0.001).

To determine whether morphine-induced hnRNPK expression was dependent on opioid receptor activation, mice were injected with vehicle or an opioid antagonist (naloxone, 10 mg/kg, s.c.) prior to morphine injection (10 mg/kg, i.v.) and the protein levels of hnRNP K in the RAIC and PAG were detected. No significant differences in hnRNP K protein level between vehicle and morphine-treated groups were observed in the RAIC (Figure 7C; *F*_2,6_ = 6.37, *P* > 0.05) and PAG (Figure 7D; *F*_2,6_ = 5.58, *P* > 0.05), indicating that naloxone prevents morphine-stimulated hnRNP K protein expression in the RAIC and PAG of mice. These findings suggest morphine induces the accumulation of hnRNP K protein via activating the opioid receptor.

### Diminishing hnRNP K expression attenuates morphine-mediated G protein-coupled inwardly rectifying potassium channel activation in AtT-20 pituitary cells

**Figure 8.**
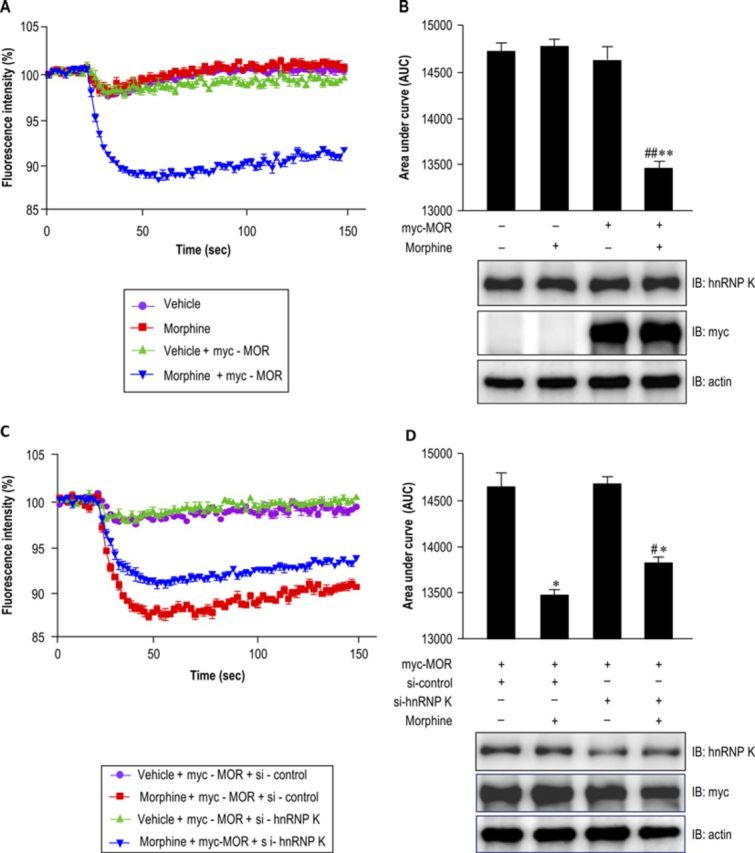
Effects of hnRNP K on morphine-mediated membrane potential hyperpolarization in pituitary AtT-20 cells. (A) Morphine induced a membrane potential hyperpolarization in MOR expressing AtT-20 cells. The pituitary AtT-20 cells were transiently transfected with vehicle or myc-MOR, the effects of morphine on membrane potential were detected in the presence or absence of morphine (1 μM) treatment. (B) Quantitative results from (A) (upper panel). The expression of hnRNP K and myc-MOR in each treatment were detected using western blotting (lower panel). **P < 0.01 versus vehicle group. ^##^*P* < 0.01 versus vehivle/myc-MOR group. (C) Silencing hnRNP K attenuated morphine-mediated membrane potential hyperpolarization. The myc-tagged MOR expressing AtT-20 cells were transiently transfected with siRNA universal negative control (si-control), or si-hnRNP K were used to perform knockdown experiments. The effects of hnRNP K on the membrane potential were evaluated using the FLIPR membrane potential assay kit in the presence or absence of morphine (1 μM) treatment (upper panel). (D) Quantitative results from (C). The expression of hnRNP K and myc-MOR in each treatment were detected using western blotting (lower panel). *P < 0.05 versus vehicle/myc-MOR/si-control group. ^#^*P* < 0.05 versus morphine/myc-MOR/si-control group. All experiments were carried out independently and at least in triplicate. The values indicate the mean ± SD. Statistical analysis was carried out using one-way ANOVA with appropriate *post hoc* tests.

Upon activation, GPCRs are coupled to trimeric G proteins, and the βγ subunit of the G protein is released from Gαβγ complex to activate the G protein-coupled inwardly rectifying potassium (GIRK) channels (22). Spinal GIRK1/GIRK2 heterotetrameric channels modulate nociception and contribute to morphine analgesia (23). The pituitary AtT-20 cell highly expressing endogenous GIRK1/GIRK2 channel is an adequate cellular model for conducting potassium channel assay (24). In this study, AtT-20 cells were transiently transfected with myc-MOR expression plasmid to detect GIRK-mediated membrane potential hyperpolarization after morphine treatment. By using the FLIPR membrane potential assay, morphine treatment altered the membrane potential in myc-tagged MOR expressing AtT-20 cells (Figure 8A). Furthermore, morphine treatment decreased 10.1% of the area under the curve of fluorescence intensity (morphine-treated group versus vehicle control group), which caused GIRK activation and altered the membrane potential in myc-tagged MOR expressing AtT-20 cells (Figure 8B, upper panel; *F*_3,8_ = 11.82, *P* < 0.01). A Newman–Keuls *post hoc* comparison revealed significant differences between the vehicle/myc-MOR and morphine/myc-MOR groups (*P* < 0.01). The protein expression levels of endogenous hnRNP K and overexpression of myc-tagged MOR in AtT-20 cells were detected using western blotting in the presence or absence of morphine (Figure 8B, lower panel). To further study the functional roles of hnRNP K in morphine-mediated MOR activation, the endogenous hnRNP K in myc-tagged MOR expressing AtT-20 cells were down-regulated by siRNA against hnRNP K (si-hnRNP K) in the presence or absence of morphine treatment, and then analyzed using FLIPR membrane potential assay. Compared with the si-control group, silencing hnRNP K attenuated morphine-mediated membrane potential hyperpolarization in myc-tagged MOR expressing AtT-20 cells (Figure 8C). Moreover, the results indicated that silencing hnRNP K attenuates morphine-mediated membrane potential hyperpolarization (Figure 8D, upper panel; *F*_3,8_ = 14.27, *P* < 0.01). A Newman–Keuls *post hoc* comparison revealed significant differences between morphine/myc-MOR/si-control and morphine/myc-MOR/si-hnRNP K groups (*P* < 0.05). No significant difference was observed between the vehicle/myc-MOR/si-control and vehicle/myc-MOR/si-hnRNP K groups (*P* > 0.05), indicating that silencing hnRNP K did not change the membrane potential in AtT-20 cells. The protein expression levels of silencing hnRNP K and overexpressing myc-tagged MOR in AtT-20 cells were detected using western blotting in the presence or absence of morphine treatment (Figure 8D, lower panel).

### Down-regulation of hnRNP K in the spinal cord of mice increases nociceptive sensitivity

**Figure 9.**
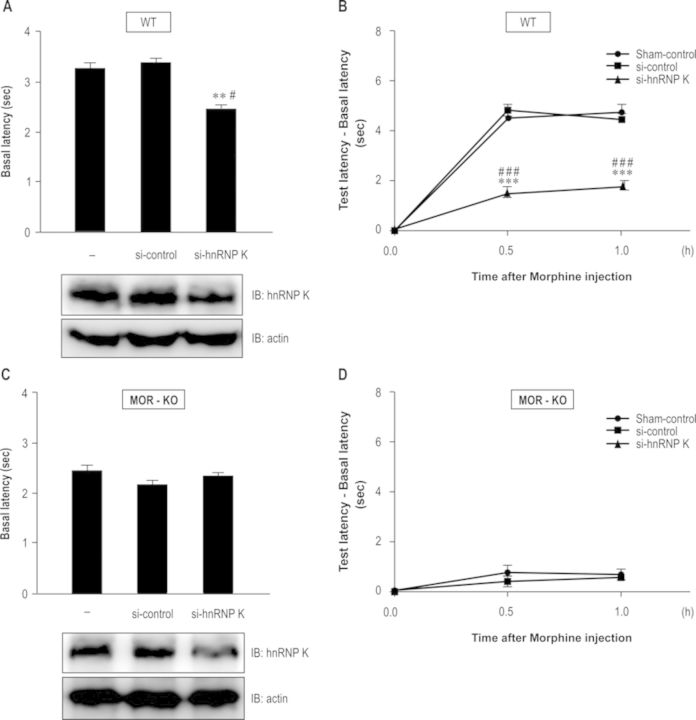
Down-regulation of hnRNP K decreased morphine-mediated analgesic effects in B6 mice. (A) Diminishing hnRNP K expression facilitated nociceptive sensitivity. Vehicle (sham-control), siRNA of negative control (si-control) or hnRNP K siRNA (si-hnRNP K) was delivered into spinal cord of WT mice by using direct *in vivo* electroporation. Three days after surgery, basal latencies of nociception were detected to estimate nociceptive sensitivity using tail-flick test (upper panel). The protein expression levels of hnRNP K in the spinal cord were detected using western blotting (lower panel). **P < 0.01 versus sham-control group. ^#^*P* < 0.05 versus si-control group. (B) Diminishing hnRNP K expression attenuated morphine-mediated analgesic effects in WT mice. After detection of basal latencies, each group of mice was injected with morphine (10 mg/kg, i.v.) to detect test latencies at the indicated time points (0.5 and 1.0 h after morphine injection) and quantitative results were calculated (test latency-basal latency). ***P < 0.001 versus sham-control group. ^###^*P* < 0.001 versus si-control group. (C) and (D) The experiments carried out in (A) and (B) were performed in MOR-KO mice. si-hnRNP K decreased its protein expression but had no effects on basal and test latencies. Statistical analysis was carried out using one-way ANOVA with appropriate *post hoc* tests.

To examine the role of hnRNP K in analgesia, we delivered hnRNP K siRNA (si-hnRNP K) to spinal cord of mice using direct *in vivo* electroporation to reduce hnRNP K expression and analyzed its influence on antinociceptive effects determined by tail-flick test. We found that hnRNP K siRNA, but not vehicle (sham-control) or siRNA of negative control (si-control), knocked down hnRNP K protein expression (Figure 9A, lower panel, analyzed by western blotting) in neurons of superficial layers of the dorsal horn, an area known to receive nociceptive inputs (Supplementary Figure S4, analyzed by immunostaining). We then measured tail-flick latencies to estimate the basal nociceptive sensitivity of these mice. The quantitative results from tail-flick test revealed that si-hnRNP K group produced 28% inhibition of basal latencies compared with si-control group (Figure 9A, upper panel; *F*_2,27_ = 6.258, *P* < 0.01). A Newman–Keuls *post hoc* comparison revealed significant differences between si-control group and si-hnRNP K group (*P* < 0.05). After detection of basal latencies, mice were injected with morphine (10 mg/kg, i.v.) and test latencies were then measured 0.5 and 1 h later. Figure 9B shows that morphine produced similar antinociceptive effects in sham-control and si-control groups. In contrast, the effects of morphine were attenuated in si-hnRNP K group (*F*_2,87_ = 133.2, *P* < 0.0001). A Newman–Keuls *post hoc* comparison revealed significant differences between si-control group and si-hnRNP K group (0.5 h, *P* < 0.001; 1.0 h, *P* < 0.001). These experiments conducted in Figure 9A and B were then repeated in MOR-KO mice. Although siRNA-mediated hnRNP K knockdown was detected (Figure 9C, lower panel), there was no difference in basal latency (Figure 9C, upper panel, *F*_2,27_ = 0.043, *P* > 0.05) or morphine-mediated analgesia (Figure 9D; *F*_2,87_ = 1.178, *P* > 0.05) among sham-control, si-control or si-hnRNP K groups in MOR-KO mice. Thus, we suggest that hnRNP K is involved in nociception and morphine-mediated analgesia thought MOR signaling.

## DISCUSSION

Opioids and their analogs are potent analgesic drugs that are widely used to treat severe pain. The MOR has been shown to play a major role in regulating these analgesic effects (25). Numerous studies have focused on this issue and their relevant therapeutic applications, but the detailed regulatory mechanisms are still unclear. In this study, we provide evidence that hnRNP K plays a functional role in pain management through MOR signaling pathway. We found potential IRESs located in the 5′ UTRs of hnRNP K transcripts. Upon morphine treatment, IRES-mediated hnRNP K translation led to hnRNP K protein accumulation in the CNS. Morphine treatment promoted hnRNP K protein accumulation, and this phenomenon could be inhibited by an opioid antagonist, naloxone, in both the RAIC and the PAG of mice. In addition, down-regulation of hnRNP K reduced morphine-mediated hyperpolarization of membrane potential in AtT-20 cells. Silencing hnRNP K expression in the mouse spinal cord increased nociceptive sensitivity and diminished morphine-stimulated analgesic effects in WT mice, but not in MOR-KO mice. Thus, our results demonstrate the translational control and biological roles of hnRNP K in analgesia through MOR-mediated signaling pathway.

Our results from mice and primary cortical neurons demonstrate that morphine treatment resulted in a dramatic up-regulation of hnRNP K protein. However, our quantitative indicate that mRNA expression levels of hnRNP K were not regulated by morphine (Figure 3A and B), suggesting that transcriptional control of hnRNP K was not involved in the morphine-mediated signaling pathway. In addition, the expression of hnRNP K was not regulated by protein stability, which differs from a previous report (8). Regulation of internal translation was first discovered in the picornavirus, which can quickly increase the number of cellular RNAs and bypass cap-dependent translation inhibition through IRES-containing mRNAs (26). Similar to the viral IRES elements, down-regulation of cap-binding factor eIF4E leads to IRES-mediated translation of cellular mRNAs under stressful conditions, such as ultraviolet-irradiation, serum starvation, hypoxia, heat shock and endoplasmic reticulum stress (27). The IRES element located in the 5′ UTR transcript contains evolutionarily conserved sequences and drives functional roles in the regulation of internal translation (12). Our results from sequence alignments indicate that a high sequence identity of hnRNP K 5′ UTRs was found between different species (Figure 4A). In addition, both human and mouse hnRNP K 5′ UTRs contained IRES sequences, which were regulated by morphine (Figure 5; Supplementary Figure S3), suggesting that hnRNP K 5′ UTR plays pivotal roles in cellular functions (Figure 4A). Furthermore, hnRNP K acts as an ITAF and facilitates hnRNP K binding with its own transcript to elevate IRES activity. This leads to protein accumulation in the CNS with morphine treatment (Figure 6). The autoregulation effects of hnRNP K can be observed in another RBP SF2/ASF, which can interact with its own transcript to reduce ribosome association (28). On the other hand, a previous report indicates that up-regulation of hnRNP K promotes IRES-mediated translation of the proto-oncogene c-myc in HeLa cells (29). However, the effect of hnRNP K on c-myc IRES activity was not observed in HEK-MOR cells (Figure 6G). Previous reports demonstrate that hnRNP K can be phosphorylated by Src family kinases, such as Lck and Src (30,31). Moreover, extracellular signal-regulated kinase 1/2-mediated phosphorylation and cytoplasmic accumulation of hnRNP K is essential for the synaptic plasticity in hippocampal neurons (32). It is likely that multiple signalling events can differentially modulate hnRNP K activity. This raises the possibility that post-translational modification, such as phosphorylation, might modulate the RNA-binding property of hnRNP K in different cells or upon different stimulations and, therefore, affect its influence on c-myc and hnRNP K IRES function.

It is identified that hnRNP K was localized in the nucleus and was mainly expressed in the developing rat CNS (33). Further, hnRNP K is a transcriptional regulator and is distributed in the nucleus of neurons, which implicates a functional role in the regulation of MOR transcription in CNS (34). In mice, we found that morphine treatment resulted in hnRNP K accumulation in the RAIC, dorsal hippocampus, PAG and dorsal horn of spinal cord (Figures 1 and Figure 7A and B), and this effect can be blocked by naloxone (Figure 7C and D). The PAG and dorsal horn of spinal cord were parts of descending nociceptive modulatory system, which was particularly important in morphine antinociception and tolerance (35–38). MOR stimulation within the RAIC and dorsal hippocampus were involved in antinociception and may played an important role of an endogenous analgesic system (39,40). The results presented here demonstrate that morphine-stimulated hnRNP K accumulation depended on activation of opioid receptors in brain regions related to antinociception. On the other hand, it has been shown that hnRNP K shuttles between the nucleus and cytoplasm via a nuclear shuttling domain and phosphorylation facilitate its cytoplasmic accumulation (41). Based on our immunofluorescence results, hnRNP K was mainly localized in the nucleus of HEK-MOR cells. However, significant cytoplasmic accumulation of hnRNP K was observed induced by morphine treatment (Figure 2A; Supplementary Figure S2). Thus, in addition to IRES-mediate protein translation, shuttling mechanisms may also contribute to cytoplasmic accumulation of hnRNP K, which warrants further investigation.hnRNP K plays important functional roles in neurons. Protein–protein interactions between hnRNP K and Hu proteins, and AU-rich elements of RBPs, have been identified as the target of p21 mRNA and are involved in neuronal differentiation through translational control (42). Moreover, hnRNP K binds to protein kinase A-responsive ribonucleic acid elements of Snap25, and plays essential roles in neuronal differentiation of rat PC12 cells (43). In addition, hnRNP K and Abi-1 synergistically modulate homeostasis of synaptic maturation and filopodia formation in the nervous system (33). It has also been shown that hnRNP K acts as a transcription factor to increase transcriptional activity for the mMOR (13). Activation of opioid receptors results in β-arrestin recruitment, inhibition of adenylyl cyclase and efflux of K^+^ through GIRK channels (44). Our results show that down-regulation of hnRNP K diminished morphine-mediated membrane potential hyperpolarization (Figure 8C). However, overexpressing HA-tagged hnRNP K cannot alter the effect of morphine on myc-tagged MOR expressing AtT-20 cells (data not shown), which may result from the dilution effect of high expression levels of endogenous hnRNP K. Interestingly, down-regulation of hnRNP K increases nociceptive sensitivity in wild-type mice, but not in MOR-KO mice, suggesting potential roles for hnRNP K in MOR-mediated analgesic effects (Figure 9A and C). In addition, silencing hnRNP K attenuates morphine-mediated tail-flick responses in WT mice, indicating that hnRNP K expression is regulated by morphine and contributes to pain management (Figure 9B). We speculate that morphine may act through hnRNP K to manipulate pain thresholds. Unraveling the role of hnRNP K in pain management might provide a method for developing strategies to treat the adverse effects of morphine, such as tolerance and hyperalgesia.

In the present study, we demonstrate a link in the nervous system between hnRNP K and morphine-mediated signaling pathways through MORs. Our findings demonstrate a novel IRES element located in the 5′ UTR of hnRNP K transcript, which promotes internal translation, protein accumulation and altered analgesic effects in response to morphine stimulation. These results demonstrate potential roles for hnRNP K in the regulation of opioid-based pain control. This study provides additional information regarding the mechanisms of opioid receptors and the signaling pathways, and therefore provides new insights into therapeutic applications for analgesia.

## Supplementary Material

SUPPLEMENTARY DATA
