# Supplementary material for: Morphine drives internal ribosome entry site-mediated hnRNP K translation in neurons through opioid receptor-dependent signaling
Source: Nucleic Acids Res. 2014 Oct 31;42(21):13012–25. doi: 10.1093/nar/gku1016 (PMC4245930; doi:10.1093/nar/gku1016)
Supplement: SUPPLEMENTARY DATA [file supp_42_21_13012__index.html]

Morphine drives internal ribosome entry site-mediated hnRNP K translation in neurons through opioid receptor-dependent signaling — Morphine drives internal ribosome entry site-mediated hnRNP K translation in neurons through opioid receptor-dependent signaling — SUPPLEMENTARY DATA 

# Morphine drives internal ribosome entry site-mediated hnRNP K translation in neurons through opioid receptor-dependent signaling

## SUPPLEMENTARY DATA

**Files in this Data Supplement:**

- SUPPLEMENTARY DATA
