# Supplementary material for: Morphine drives internal ribosome entry site-mediated hnRNP K translation in neurons through opioid receptor-dependent signaling
Source: Nucleic Acids Res. 2014 Oct 31;42(21):13012–25. doi: 10.1093/nar/gku1016 (PMC4245930; doi:10.1093/nar/gku1016)
Supplement: SUPPLEMENTARY DATA [file supp_gku1016_nar-03662-y-2013-File011.pdf]

Supplementary Data

Supplementary Figure 1. Immunostaining of astrocytes and hnRNP K-positive cells after injection of morphine. Immunostaining for hnRNP K (red) and GFAP (green) 1 h after injection of morphine (10 mg/kg, i.v.). DAPI (blue) is the nuclear marker. Scale bars, 50 μm.

Supplementary Figure 2. Morphine up-regulated nuclear and cytoplasmic hnRNP K protein expression in HEK-MOR cells. The HEK-MOR cells were treated with vehicle or morphine (1 μM). Cell extracts were separated into cytoplasmic and nuclear fractions and subjected to western blot analysis (upper panel). Quantification of protein expression was measured using densitometry (lower panel). All experiments were carried out independently and at least in triplicate. The values indicate the mean ± SD. * $p < 0.05$, ** $p < 0.01$ vs. vehicle control group, Student's *t*-test.

Supplementary Figure 3. The 5′ UTR of mouse hnRNP K contains IRES function, and its IRES activities were regulated by morphine. (A) The diagram of phpRmKF bicistronic reporter construct. The 5′ UTR of mouse hnRNP K (GenBank accession number NM_025279) was cloned to generate the phpRmKF reporter construct. (B) The 5′ UTR of mouse hnRNP K shows IRES activity. The indicated three reporter plasmids were transiently transfected into HEK-MOR cells. The ratio of (Firefly/Renilla) was analyzed by reporter assay ($F_{2,6} = 53.51$, $p < 0.001$). *** $p < 0.001$ vs. phpRF group. (C) After transfection, the mRNA ratio (Firefly/Renilla) of three constructs was analyzed by quantitative real-time RT-PCR ($F_{2,6} = 3.72$, $p > 0.05$). (D) Morphine enhanced IRES activity of hnRNP K. The indicated three reporter plasmids were transiently transfected into HEK-MOR cells, and treated with vehicle or morphine (1 μM) and harvested at the indicated time points (1 h). The ratio of Firefly/Renilla was analyzed by

reporter assay. ** $p < 0.01$ vs. vehicle control group. All experiments were carried out independently and at least in triplicate. The values indicate the mean ± SD. Statistical analysis was carried out using one-way ANOVA with appropriate *post hoc* tests (B) and (C), or Student's *t*-test (D).

Supplementary Figure 4. Down-regulation of hnRNP K in spinal cord dorsal horn neurons. The siRNA (si-control or si-hnRNP K) was transfected into spinal cord of B6 mice. Three days later, the B6 mice were injected with vehicle or morphine (10 mg/kg, i.v.) and sacrificed at the indicated time point (60 min). The expression levels of hnRNP K (red) and NeuN (green) were visualized using immunostaining. DAPI (blue) is the nuclear marker. Scale bars, 50 μm.

Supplementary Figure 1

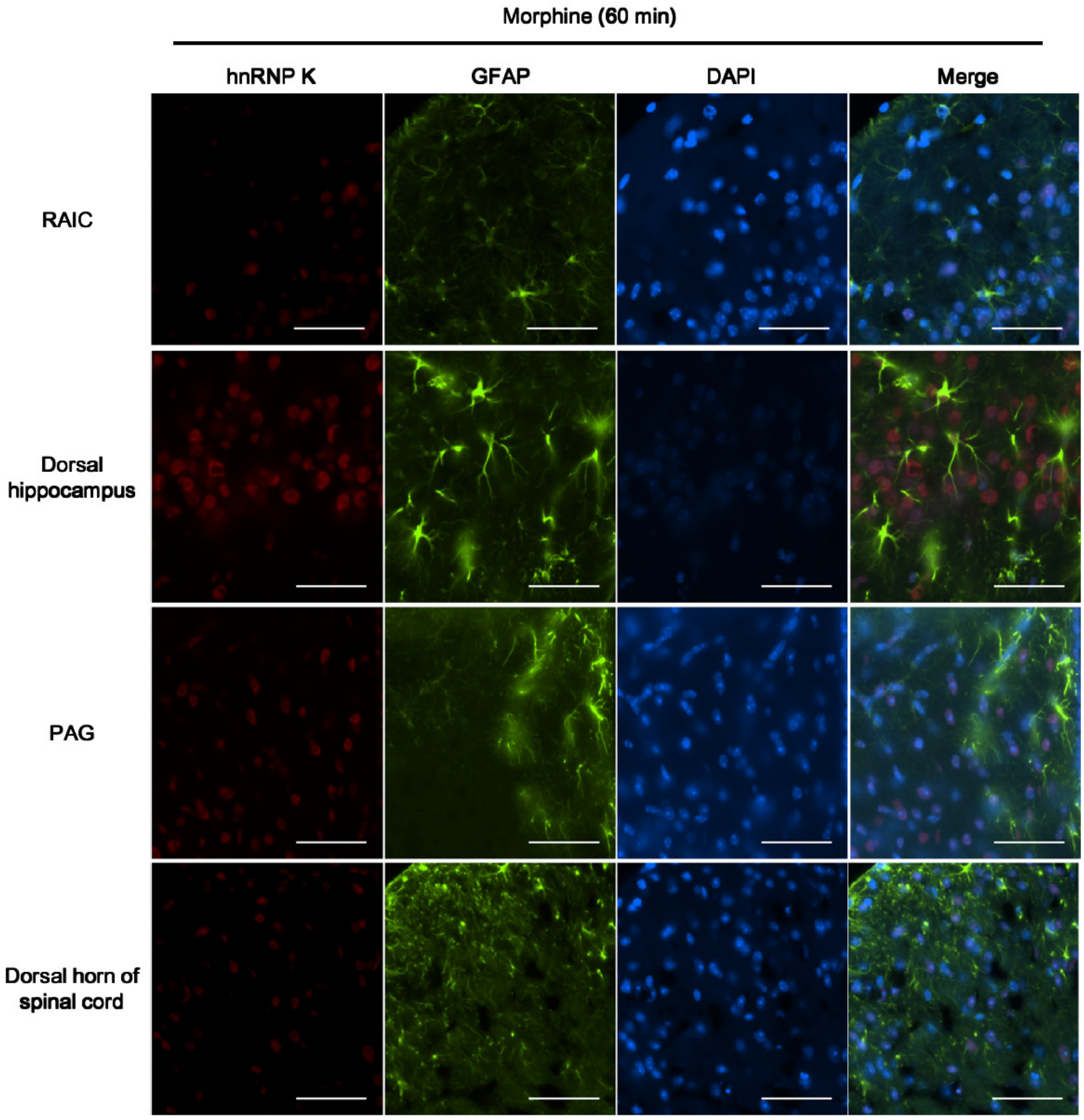

Supplementary Figure 2

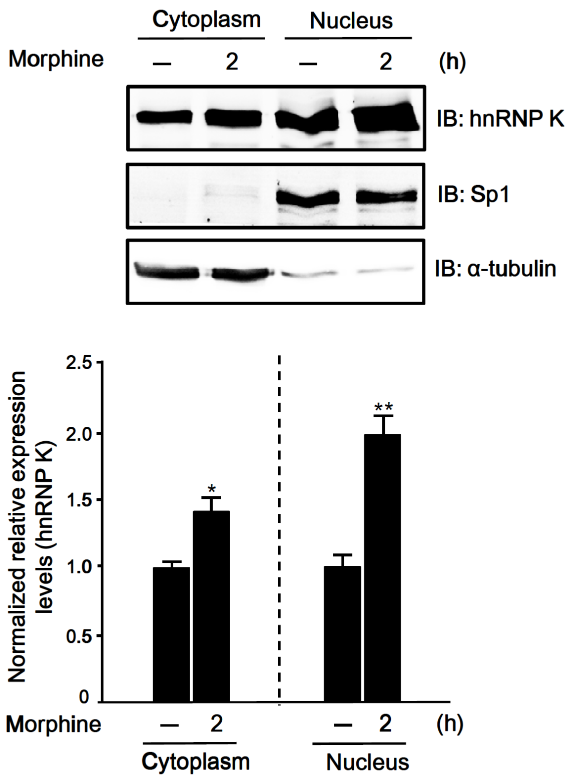

Supplementary Figure 3

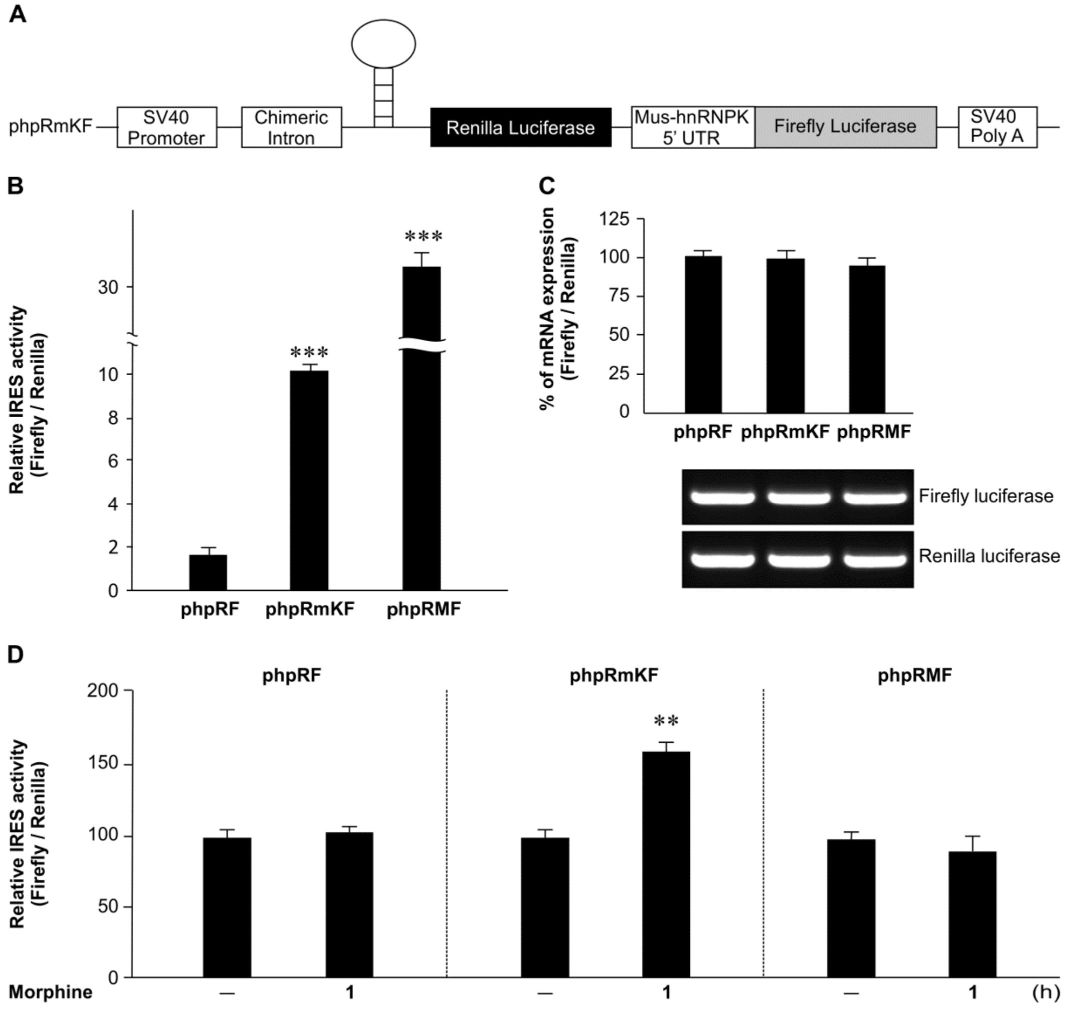

Supplementary Figure 4

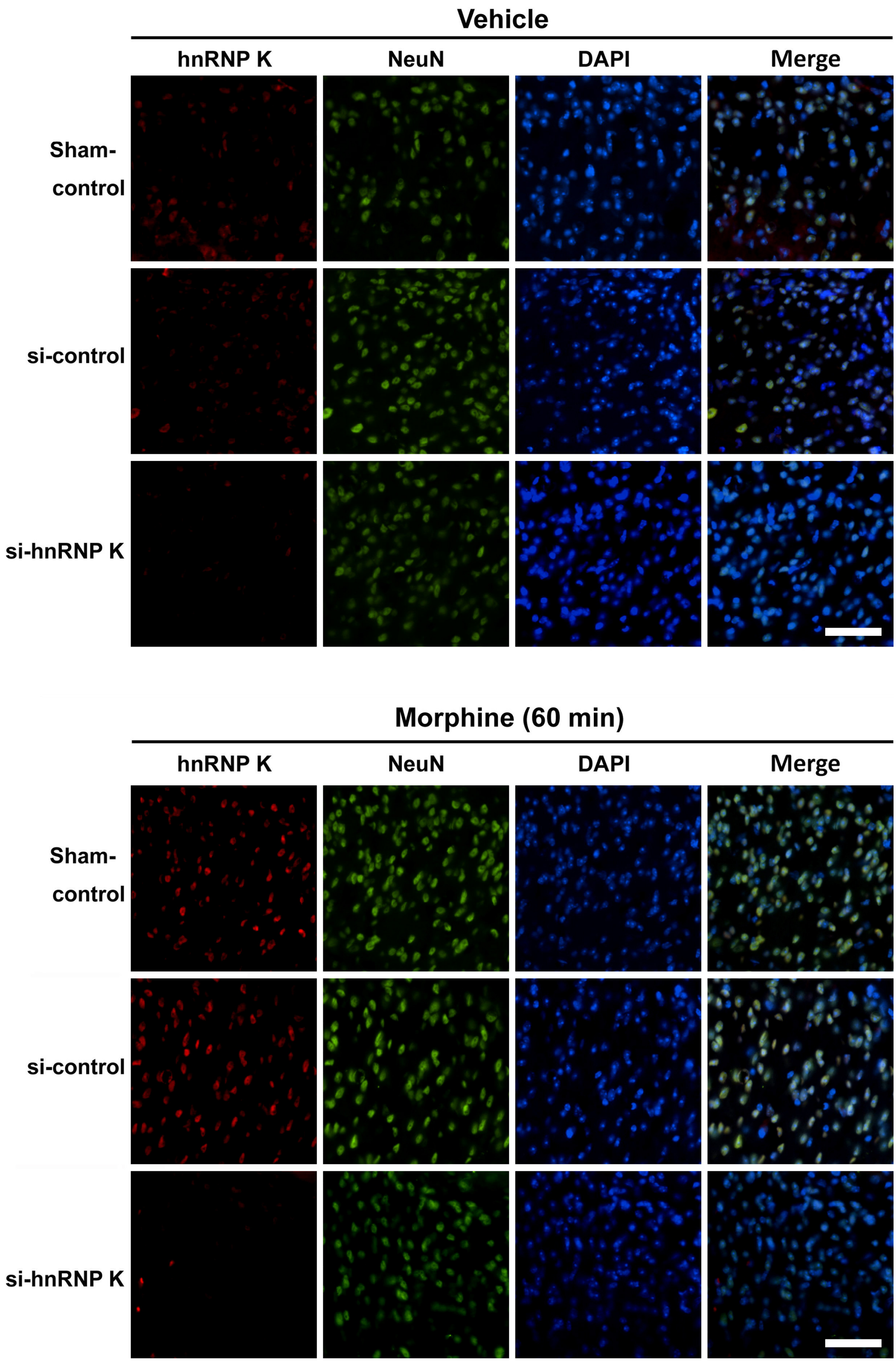

Supplementary Table 1. Primer sequences used for PCR amplification and plasmid constructions

Gene	Accession No.		Primer (5' → 3')	RE	Amplicon (bp)
5' UTR of human hnRNP K-V1	NM_002140.3	Sense	GACTAGTCCCCTAGCCGCCCCTCCCCCCAGCT	*Spe*I	259
		Antisense	CATGCCATGGCATGATTCTTTTATTAAACGGGCAC	*Nco*I	
5' UTR of human hnRNP K-V2	NM_031263.2	Sense	GACTAGTCTAGGGCGCGACGGCGGGGAGGACG	*Spe*I	224
		Antisense	CATGCCATGGCATGATTCTTTTATTAAACGGGCAC	*Nco*I	
5' UTR of human hnRNP K-V1	NM_002140.3	Sense	CCCAAGCTTGGGCCCTAGCCGCCCCTCCCCCCAGCT	*Hind*III	259
		Antisense	CATGCCATGGCATGATTCTTTTATTAAACGGGCAC	*Nco*I	
5' UTR of human hnRNP K-V2	NM_031263.2	Sense	CCCAAGCTTGGGTAGGGCGCGACGGCGGGGAGGACG	*Hind*III	224
		Antisense	CATGCCATGGCATGATTCTTTTATTAAACGGGCAC	*Nco*I	
5' UTR of human nucleolin	NM_005381.2	Sense	GACTAGTCCTTTCGCCTCAGTCTCGAGCTCTC	*Spe*I	141
		Antisense	CATGCCATGGCATGGATGGCGGCGGAGTGTGAAGCGGA	*Nco*I	
5' UTR of mouse hnRNP K	NM_025279.2	Sense	GACTAGTCACAGAGTGCGCGAACGAGAAAGGA	*Spe*I	151
		Antisense	CATGCCATGGCATGTTTCTTGAATTAAATGGATCCACC	*Nco*I	
CDS of human hnRNP K	NM_031263.2	Sense	GGAATTCCGAAACTGAACAGCCAGAAGAAACC	*EcoR*I	1394
		Antisense	CCGCTCGAGCGGGAATCCTTCAACATCTGCATACTG	*Xho*I	

❖ The abbreviations : RE, restriction enzyme site; hnRNP K,heterogeneous nuclear ribonucleoprotein K; UTR, untranslated region; V1, transcript variant 1; V2, transcript variant 2; bp, base pairs; CDS, coding region.

❖ The underlined nucleotides are restriction enzyme sites included in the primers.

Supplementary Table 2. Primer sequences used for real-time PCR amplification

Gene	Accession No.	Primer (5' → 3')		Amplicon (bp)
hnRNPK	NM_002140.3	Sense	TTCAGTCCCAGACAGCAGTG	165
		Antisense	TCCACAGCATCAGATTCGAG	
Nucleolin	NM_005381.2	Sense	TCATGGTGAAGCTCGCGAAGGCAG	511
		Antisense	ATTTCATCTTCATCCTCATCCTCG	
GAPDH	NM_002046.5	Sense	CCCACTCCTCCACCTTTGAC	186
		Antisense	TCTCTCTTCCTCTTGTGCTCTTG	
Firefly luciferase		Sense	GGTTCCATCTGCCAGGTATCAGG	300
		Antisense	CGTCTTCGTCCCAGTAAGCTATG	
Renilla luciferase		Sense	AAAGGTGAAGTTCGTCGTCCAAC	300
		Antisense	TTTGAGAACTCGCTCAACGAACG	

❖ The abbreviations : hnRNP K, heterogeneous nuclear ribonucleoprotein K; GAPDH, glyceraldehyde-3-phosphate dehydrogenase; bp, base pairs.
